# Dataset on synthesis and crystallographic structure of phenyl(TMP)iodonium(III) acetate

**DOI:** 10.1016/j.dib.2019.104063

**Published:** 2019-05-25

**Authors:** Hideyasu China, Daichi Koseki, Kazuki Samura, Kotaro Kikushima, Yasuko In, Toshifumi Dohi

**Affiliations:** a College of Pharmaceutical Science, Ritsumeikan University, 1-1-1 Nojihigashi, Kusatu, Shiga, 525-8577, Japan; b Osaka University of Pharmaceutical Science, 4-20-1 Nasahara, Takatsuki, Osaka, 569-1094, Japan

**Keywords:** Hypervalent iodine compound, Iodonium(III) salt, Acetate ligand, Two geometrical states

## Abstract

The data in this article are related to research article ‘‘Efficient *N*-arylation of azole compounds utilizing selective aryl-transfer TMP-iodonium (III) reagents (Koseki et al., 2019). For the title compound, phenyl(2,4,6-trimethoxyphenyl)iodonium(III) acetate (Ph(TMP)IOAc), the single-crystal X-ray diffraction measurement together with NMR analysis, like also the method of synthesis and crystallization are presented. The X-ray structure analysis has revealed that the two types of geometries regarding the acetate anion attached to phenyl (TMP)iodonium (III) cation are found in the crystal states.

**Table:** Specifications table

Subject area	*Chemistry*
More specific subject area	*Organic Chemistry, Reagent for Coupling Reaction*
Type of data	*Figures, tables, text file. x-ray (figures, tables),*^*1*^*H and*^*13*^*C NMRs (figures, text), synthesis (text)*
How data was acquired	X-ray (X-ray crystallographic analysis was performed on a HPC diffractometer (Rigaku XtaLAB P200)). NMR (JEOL ECS 400 NMR spectrometer, solvent CDCl_3_),
Data format	*X-ray (analyzed), NMR (raw)*
Experimental factors	*Crystallization at room temperature. phenyl(TMP)iodonium(III) acetate - chloroform/hexane mixture (2/5), CDCl*_*3*_*in NMR tube.*
Experimental features	*Protection from light during recrystallization is required.*
Data source location	*City: Kusatsu, Country: Japan, Latitude: N34°58′46.6″, Longitude: E135°57′46.7″, (Lat,Long: 34.979604, 135.962984), City: Takatsuki, Country: Japan, Latitude:* N34°51′51.7″*, Longitude: E135°34′28.1″, (Lat,Long: 34.864362, 135.574469),*
Data accessibility	*The Cambridge Crystallographic Data Centre no. CCDC 1555121 (*http://www.ccdc.cam.ac.uk/conts/retrieving.html*, email:**deposit@ccdc.cam.ac.uk**.).*
Related research article	*Daichi Koseki, Erika Aoto, Toshitaka Shoji, Kazuma Watanabe, Yasuko In, Yasuyuki Kita, Toshifumi Dohi, Efficient N-arylation of azole compounds utilizing selective aryl-transfer TMP-iodonium(III) reagents, Tetrahedron Letters**[1]*

**Table:** 

**Value of the data** • The X-ray structural information for phenyl(2,4,6-trimethoxyphenyl)iodonium (III) acetate (Ph(TMP)IOAc) presented in this work is the first data for an organic iodonium (III) salt with two geometrical states in a crystal. • Convenient synthetic method for preparing Ph(TMP)IOAc with high purity, which is applicable to the synthesis of other analogues is presented. • Our dataset is useful for organic chemists and physicists who study organic hypervalent iodine compounds. • The structural data of Ph(TMP)IOAc has additional value as an important intermediate in the metal-free esterification reactions.

## 1 Data

Recently, utilization of the auxiliary and the dummy ligand in diaryliodonium (III) salts for a selective aryl-transfer has been actively investigated after the discovery of Mes-iodonium (III) salts (Mes = mesityl) [2], [3]. The organic salts consisting of phenyl (TMP)iodonium (III) cation (TMP = 2,4,6-trimethoxyphenyl) and the counterion, such as Cl^−^ [4], [5], Br^−^ [4], BF_4_^−^ [4], TfO^−^ [4], [6], TsO^−^ [7], and CF_3_COO^−^ [8], serve as efficient aryl-transfer reagents for metal-free coupling reactions [9], [10], [11]. In our work, the aryl (TMP)iodonium (III) salts were applied as the efficient arylating agents for the copper-catalyzed *N*-arylation of azole compounds, which turned out that these iodonium (III) salts have high reactivities even in the metal-catalyzed coupling together with the reported exclusive aryl-group transfer behavior [1]. Therefore, the synthesis and structural information for Ph(TMP)IOAc are very important. The first example of the X-ray structural analysis is worth to notice. Our original method for preparation of the diaryliodonium (III) salts [12] enables to obtain the studied compound of high purity suitable for single-crystal growth (see Table 1, Table 2, Table 3, Table 4).

**Table 1:** X-ray experimental details for Ph(TMP)IOAc.

Crystal data	
Chemical formula	C_17_H_19_IO_5_
*Mw*	430.22
Crystal system, space group	Orthorhombic, *Pbca*
Temperature (K)	120
*a*, *b*, *c* (Å)	15.7731 (1), 12.6253 (1), 17.1040 (2)
*V* (Å^3^)	3406.09 (5)
*Z*	8
Radiation type	Cu *K*α
μ (mm^−1^)	14.98
Crystal size (mm)	0.46 × 0.26 × 0.13
**Data collection**	
Diffractometer	X-ray crystallographic analysis was performed on a HPC diffractometer (Rigaku XtaLAB P200).
Absorption correction	Multi-scan *CrysAlis PRO* 1.171.39.20a (Rigaku Oxford Diffraction, 2015) Empirical absorption correction using spherical harmonics, implemented in SCALE3 ABSPACK scaling algorithm.
*T*_min_, *T*_max_	0.111, 1.000
No. of measured, independent and observed [*I* > 2σ(*I*)] reflections	3456, 3456, 3230
*R*_int_	0.106
(sin θ/λ)_max_ (Å^−1^)	0.625
**Refinement**	
*R* [*F*^2^ > 2σ(*F*^2^)], w*R* (*F*^2^), *S*	0.056, 0.156, 1.09
No. of reflections	3456
No. of parameters	208
H-atom treatment	H-atom parameters constrained
Δρ_max_, Δρ_min_ (e Å^−3^)	2.50, −3.39

Computer programs: *CrysAlis PRO* 1.171.39.3a (Rigaku OD, 2015), SHELXT-2014/5 (Sheldrick, 2014), *SHELXL2014*/7.

**Table 2:** Selected bond lengths (Å) of Ph(TMP)IOAc.

I1A—C1B	2.085 (5)	C9B–O3B	1.425 (6)
I1A—C1C	2.130 (5)	C9B–H9B1	0.9600
C1B–C2B	1.398 (7)	C9B–H9B2	0.9600
C1B–C6B	1.408 (6)	C9B–H9B3	0.9600
C2B–O1B	1.363 (5)	C1C–C2C	1.372 (7)
C2B–C3B	1.392 (7)	C1C–C6C	1.372 (7)
C3B–C4B	1.392 (7)	C2C–C3C	1.396 (7)
C3B–H3B	0.9300	C2C–H2C	0.9300
C4B–O2B	1.353 (6)	C3C–C4C	1.381 (9)
C4B–C5B	1.395 (7)	C3C–H3C	0.9300
C5B–C6B	1.384 (6)	C4C–C5C	1.394 (9)
C5B–H5B	0.9300	C4C–H4C	0.9300
C6B–O3B	1.359 (5)	C5C–C6C	1.382 (8)
C7B–O1B	1.431 (6)	C5C–H5C	0.9300
C7B–H7B1	0.9600	C6C–H6C	0.9300
C7B–H7B2	0.9600	C1D—O2D	1.237 (6)
C7B–H7B3	0.9600	C1D—O1D	1.267 (6)
C8B–O2B	1.447 (7)	C1D—C2D	1.514 (7)
C8B–H8B1	0.9600	C2D—H2D1	0.9600
C8B–H8B2	0.9600	C2D—H2D2	0.9600
C8B–H8B3	0.9600	C2D—H2D3	0.9600

**Table 3:** Selected torsion angles (°) of Ph(TMP)IOAc.

C6B–C1B–C2B–O1B	−179.3 (4)	I1A—C1B–C6B–C5B	178.0 (3)
I1A—C1B–C2B–O1B	3.4 (5)	C3B–C2B–O1B–C7B	−0.8 (7)
C6B–C1B–C2B–C3B	−0.3 (6)	C1B–C2B–O1B–C7B	178.1 (4)
I1A—C1B–C2B–C3B	−177.6 (3)	C3B–C4B–O2B–C8B	−0.6 (7)
O1B–C2B–C3B–C4B	179.3 (4)	C5B–C4B–O2B–C8B	179.1 (4)
C1B–C2B–C3B–C4B	0.5 (7)	C5B–C6B–O3B–C9B	−1.7 (6)
C2B–C3B–C4B–O2B	178.6 (4)	C1B–C6B–O3B–C9B	179.1 (4)
C2B–C3B–C4B–C5B	−1.0 (7)	C6C–C1C–C2C–C3C	0.3 (8)
O2B–C4B–C5B–C6B	−178.3 (4)	I1A—C1C–C2C–C3C	−177.8 (4)
C3B–C4B–C5B–C6B	1.4 (6)	C1C–C2C–C3C–C4C	0.5 (9)
C4B–C5B–C6B–O3B	179.7 (4)	C2C–C3C–C4C–C5C	0.1 (9)
C4B–C5B–C6B–C1B	−1.2 (6)	C3C–C4C–C5C–C6C	−1.4 (10)
C2B–C1B–C6B–O3B	179.9 (4)	C2C–C1C–C6C–C5C	−1.6 (8)
I1A—C1B–C6B–O3B	−2.8 (5)	I1A—C1C–C6C–C5C	176.5 (5)
C2B–C1B–C6B–C5B	0.7 (6)	C4C–C5C–C6C–C1C	2.2 (10)

**Table 4:** Selected bond angles (°) of Ph(TMP)IOAc.

C1B–I1A—C1C	91.08 (16)	O3B–C9B–H9B3	109.5
C2B–C1B–C6B	119.1 (4)	H9B1–C9B–H9B3	109.5
C2B–C1B–I1A	120.2 (3)	H9B2–C9B–H9B3	109.5
C6B–C1B–I1A	120.6 (3)	C2B–O1B–C7B	118.5 (4)
O1B–C2B–C3B	123.1 (4)	C4B–O2B–C8B	117.4 (4)
O1B–C2B–C1B	115.6 (4)	C6B–O3B–C9B	117.6 (4)
C3B–C2B–C1B	121.3 (4)	C2C–C1C–C6C	121.9 (5)
C4B–C3B–C2B	118.0 (4)	C2C–C1C–I1A	119.0 (4)
C4B–C3B–H3B	121.0	C6C–C1C–I1A	119.1 (4)
C2B–C3B–H3B	121.0	C1C–C2C–C3C	119.0 (5)
O2B–C4B–C3B	123.7 (5)	C1C–C2C–H2C	120.5
O2B–C4B–C5B	114.1 (4)	C3C–C2C–H2C	120.5
C3B–C4B–C5B	122.2 (4)	C4C–C3C–C2C	120.3 (5)
C6B–C5B–C4B	118.9 (4)	C4C–C3C–H3C	119.8
C6B–C5B–H5B	120.6	C2C–C3C–H3C	119.8
C4B–C5B–H5B	120.6	C3C–C4C–C5C	119.1 (5)
O3B–C6B–C5B	124.1 (4)	C3C–C4C–H4C	120.4
O3B–C6B–C1B	115.3 (4)	C5C–C4C–H4C	120.4
C5B–C6B–C1B	120.5 (4)	C6C–C5C–C4C	120.8 (5)
O1B–C7B–H7B1	109.5	C6C–C5C–H5C	119.6
O1B–C7B–H7B2	109.5	C4C–C5C–H5C	119.6
H7B1–C7B–H7B2	109.5	C1C–C6C–C5C	118.8 (5)
O1B–C7B–H7B3	109.5	C1C–C6C–H6C	120.6
H7B1–C7B–H7B3	109.5	C5C–C6C–H6C	120.6
H7B2–C7B–H7B3	109.5	O2D—C1D—O1D	125.4 (4)
O2B–C8B–H8B1	109.5	O2D—C1D—C2D	118.9 (4)
O2B–C8B–H8B2	109.5	O1D—C1D—C2D	115.6 (4)
H8B1–C8B–H8B2	109.5	C1D—C2D—H2D1	109.5
O2B–C8B–H8B3	109.5	C1D—C2D—H2D2	109.5
H8B1–C8B–H8B3	109.5	H2D1—C2D—H2D2	109.5
H8B2–C8B–H8B3	109.5	C1D—C2D—H2D3	109.5
O3B–C9B–H9B1	109.5	H2D1—C2D—H2D3	109.5
O3B–C9B–H9B2	109.5	H2D2—C2D—H2D3	109.5
H9B1–C9B–H9B2	109.5		

**Scheme 1:**
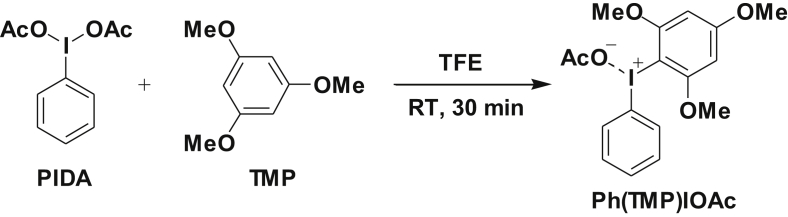
Direct synthesis of Ph(TMP)IOAc by the reaction of PIDA with TMP.

**Fig. 1:**
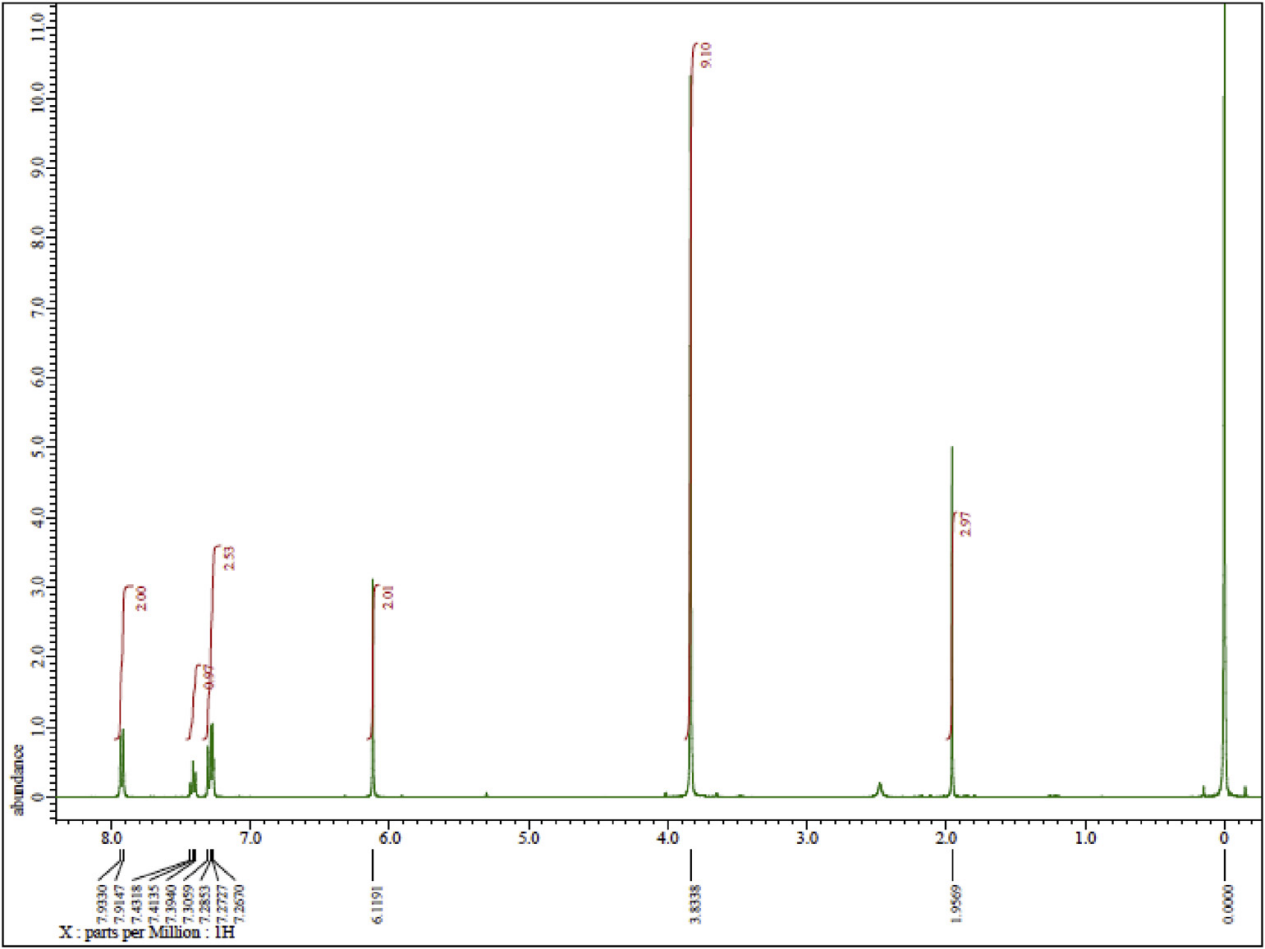
^1^H NMR spectrum of Ph(TMP)IOAc.

Ph(TMP)IOAc was synthesized by direct condensation between phenyliodine (III) diacetate (PIDA) and 1,3,5-trimethoxybenzene (TMP) in fluoroalcohol medium under mild conditions (Scheme 1). The structure of Ph(TMP)IOAc was determined by two-dimensional NMR analyses (Fig. 3, Fig. 4). The ^1^H NMR spectrum in Fig. 1 supports the high purity of Ph(TMP)IOAc obtained in this study. X-ray structural analysis have suggested that two geometrical states for Ph(TMP)IOAc appear in a crystal in the three-dimensional structure (Fig. 5, Fig. 6).

**Fig. 2:**
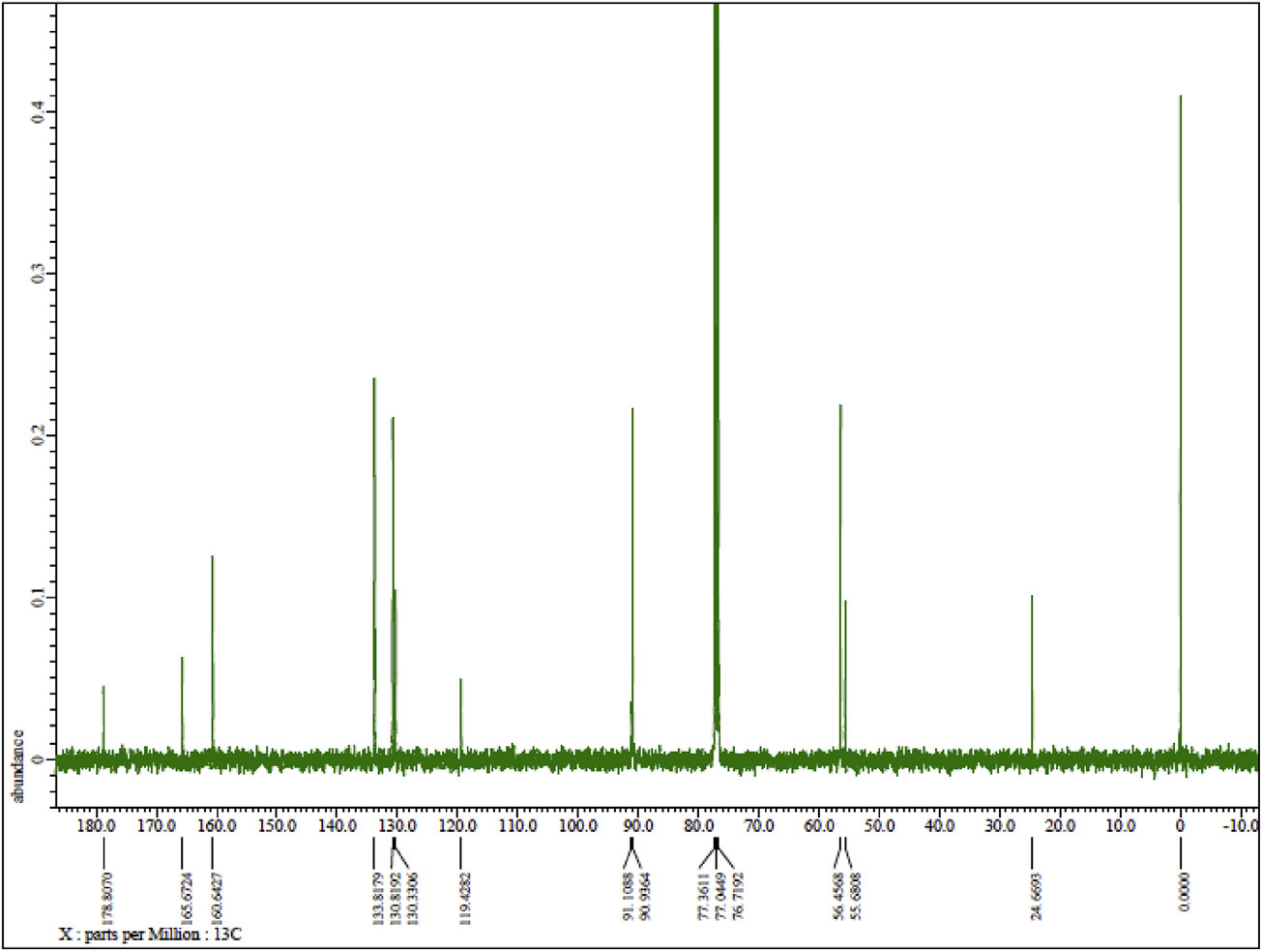
^13^C NMR spectrum of Ph(TMP)IOAc.

**Fig. 3:**
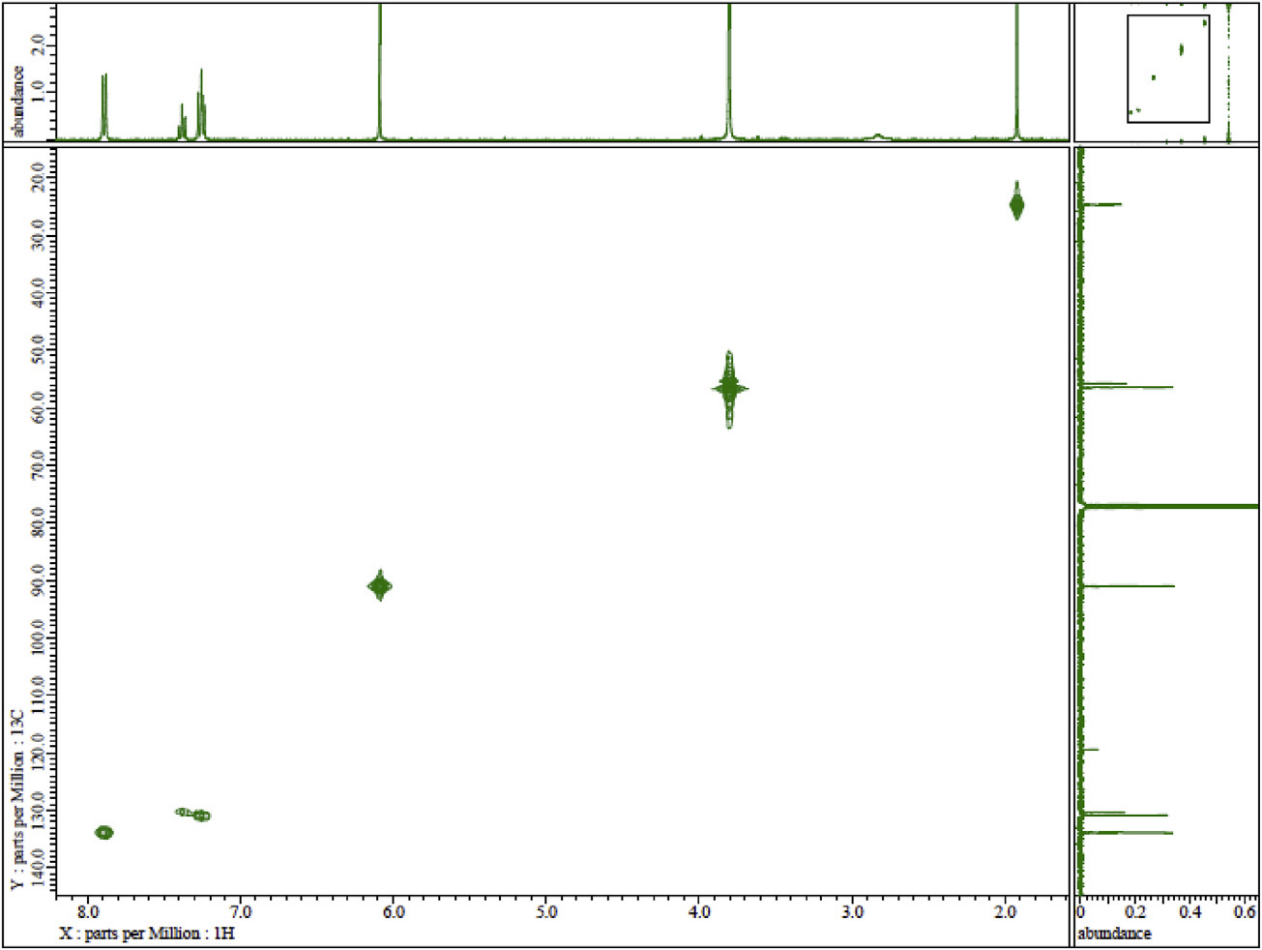
HMQC spectrum of Ph(TMP)IOAc.

**Fig. 4:**
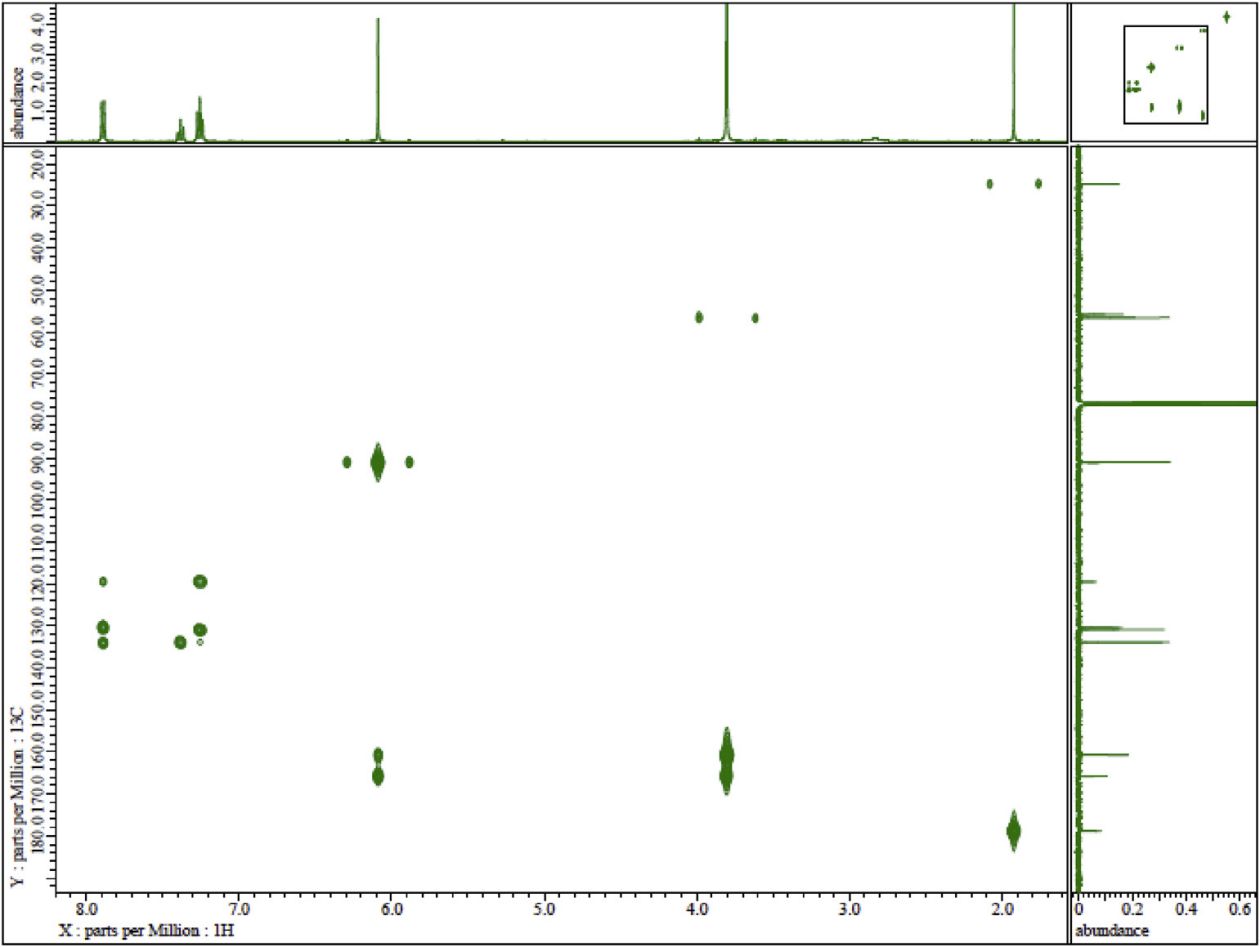
HMBC spectrum of Ph(TMP)IOAc.

**Fig. 5:**
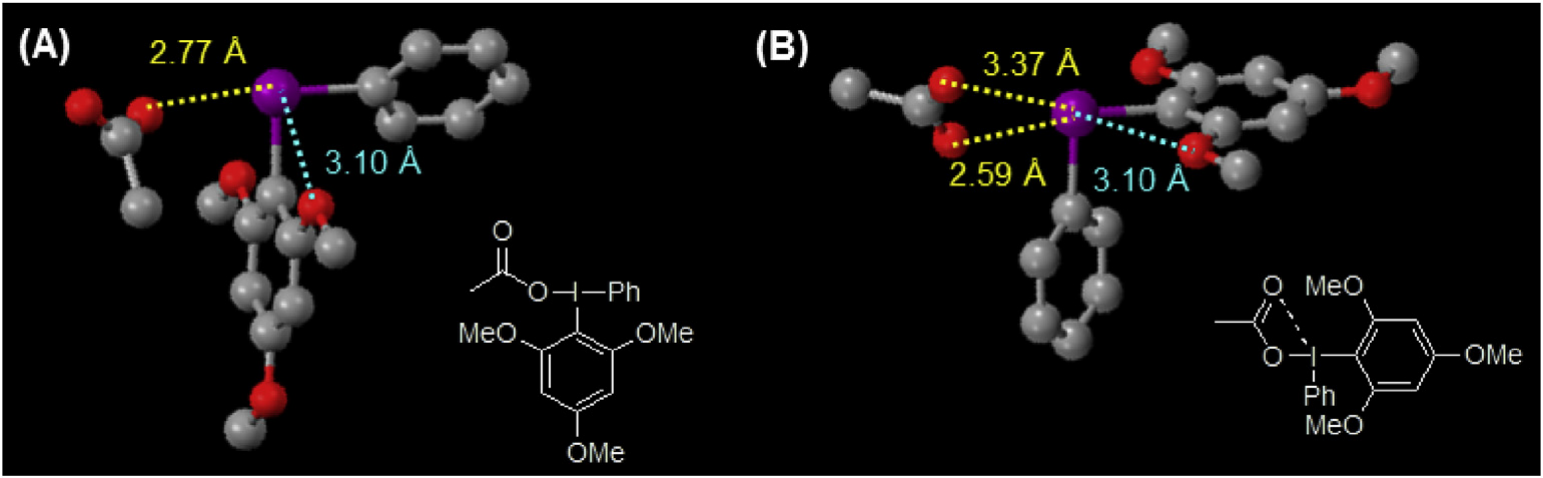
The two geometrical states of Ph(TMP)IOAc present in a crystal.

**Fig. 6:**
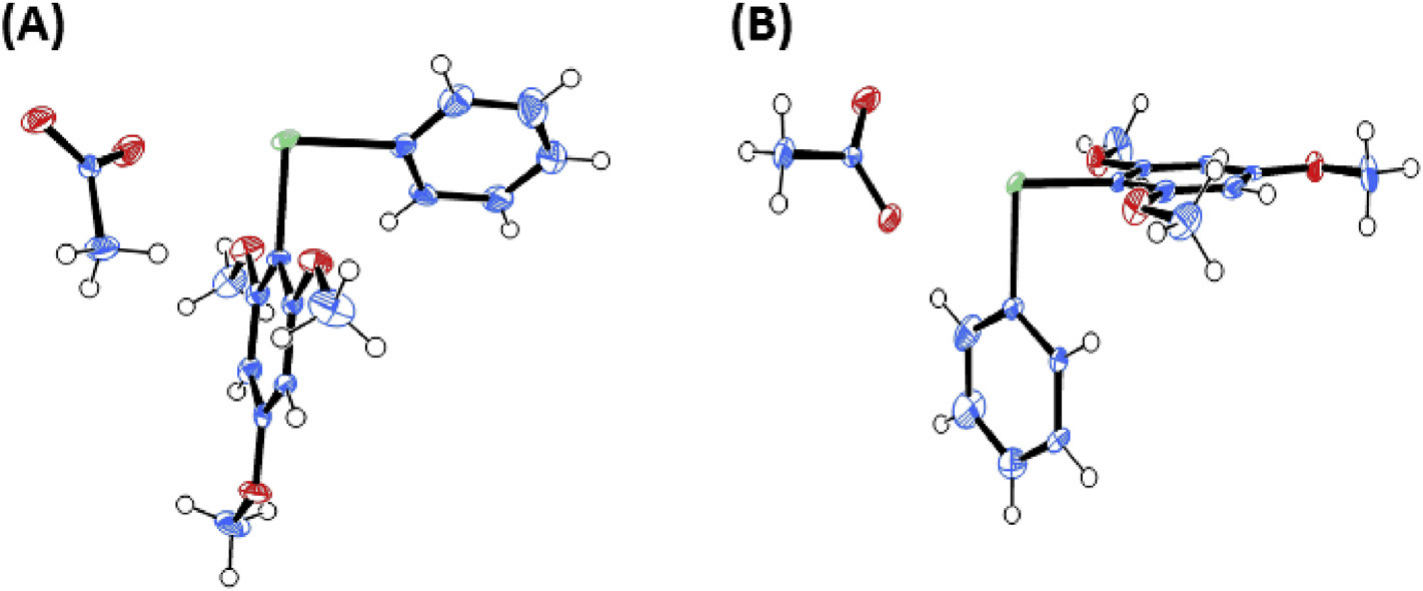
ORTEP drawings for the two states of Ph(TMP)IOAc.

## 2 Experimental design, materials, and methods

### 2.1 Materials

The solvents, starting materials, and reagents were purchased from Nacalai tesque and Tokyo Chemical Industry CO. Ltd.

### 2.2 Synthesis of Ph(TMP)IOAc

Ph(TMP)IOAc was prepared according to our reported procedure [12]. Thus, to a solution of 1,3,5-trimethoxybenzene (TMP, 168 mg, 1.0 mmol) in 2,2,2-trifluoroethanol (TFE, 2 mL) was added phenyliodine (III) diacetate (PIDA, 322 mg, 1.0 mmol) at once. The mixture was stirred at room temperature for 30 min, and the solvent was then removed by evaporation. To the residue was added diethyl ether (20 mL) for precipitation of the Ph(TMP)IOAc and the resulting suspension was then allowed to stand for 2 h. The precipitate was filtered followed by washing with diethyl ether and dried to afford Ph(TMP)IOAc (350 mg, 0.81 mmol). Yield 81%. White powder. Melting point 121.8 (121.5–122.1) °C.

### 2.3 General information for NMR analyses

The ^1^H and ^13^C NMR spectra were recorded on an ECS 400 NMR spectrometer (JEOL Ltd., Tokyo, Japan) at 400 MHz and 100 MHz, respectively, using CDCl_3_ as the solvent. The chemical shifts (*δ*) are expressed in ppm relative to tetramethylsilane (TMS) as an internal standard. Coupling constants (*J*) are expressed in Hz. Signal multiplicities are represented as singlet (s), doublet (d), and triplet (t). Assignments of the proton and carbon positions in the compound were performed by PFG-HMQC and PFG-HMBC analyses.

### 2.4 NMR

JEOL ECS 400 NMR spectrometer, solvent CDCl_3_, TMS standard. Concentration: 13 mg in 0.75 mL (Fig. 1, Fig. 2, Fig. 3, Fig. 4). ^1^H NMR (400 MHz, CDCl_3_): *δ* 1.95 (3H, s, CH_3_CO), 3.83 (9H, s, OMe), 6.12 (2H, s, *m-*TMP), 7.29 (2H, t, *J* = 7.8 Hz, *m*-Ph), 7.41 (1H, t, *J* = 7.8 Hz, *p*-Ph), 7.92 (2H, d, *J* = 8.2 Hz, *o*-Ph). ^13^C NMR (100 MHz, CDCl_3_): *δ* 24.6 (CH_3_COO), 55.7 (*p*-OMe), 56.5 (*o*-OMe), 90.9 (*m*-TMP), 91.0 (*ipso*-TMP), 119.4 (*ipso*-Ph), 130.3 (*p*-Ph), 130.8 (*m*-Ph), 133.8 (*o*-Ph), 160.6 (*o-*TMP), 165.7 (*p-*TMP), 178.8 (CH_3_COO).

### 2.5 Crystallization

The crystals were obtained at room temperature from chloroform/hexane mixture under a shading condition. Ph(TMP)IOAc was dissolved in chloroform and the insoluble material was removed by filtration. Hexane was added to the filtrate in sample bottle to reach the chloroform/hexane ratio 2/5. After standing for 1 day, the several crystals suitable for the X-ray structural analysis were obtained.

### 2.6 X-ray

The single-crystal X-ray diffraction experiment was performed on the HPC diffractometer (Rigaku XtaLAB P200)). The two types of geometries for Ph(TMP)IOAc in a crystal state are shown in Fig. 5. In Fig. 5(A), it was found that the distance between the iodine atom in the cation and an oxygen atom in the anion is 2.77 Å. On the other hand, the distances between the iodine atom in the cation and oxygen atoms in the anion were 2.59 Å and 3.37 Å, respectively (Fig. 5(B)). In both geometries, the distances between the iodine atom in the cation and two oxygen atoms in the methoxy group were 3.10 Å (Fig. 5(A) and (B)).
