# Supplementary material for: Dataset on synthesis and crystallographic structure of phenyl(TMP)iodonium(III) acetate
Source: Data Brief. 2019 May 25;25:104063. doi: 10.1016/j.dib.2019.104063 (PMC6562192; doi:10.1016/j.dib.2019.104063)
Supplement: Multimedia component 1 [file mmc1.zip › dib_104063_COI_mmc1.PDF]

Conflicts of Interest: COI

The authors have no conflicts of interest directly relevant to the content of the following article;

Synthesis and crystallographic structure of phenyl(TMP)iodonium(III) acetate

Hideyasu China, Daichi Koseki, Kazuki Samura, Kotaro Kikushima,

Yasuko In,* Toshifumi Dohi*

Corresponding Author

Toshifumi Dohi, Ph.D.

Professor

College of Pharmaceutical Sciences, Ritsumeikan University

1-1-1 Nojihigashi, Kusatsu, Shiga 525-8577, Japan

Tel. +81-77-561-4908

E-mail: td1203@ph.ritsumei.ac.jp

Name: Toshifumi Dohi Toshifumi Dohi, Professor

Date May 15th, 2019
